# Preparation and Use of Iron on Carbon Foam for Removal of Organic Dye from Water: Batch Studies

**DOI:** 10.3390/ma16196350

**Published:** 2023-09-22

**Authors:** Siphesihle Praise-God Khumalo, David Lokhat, Ajay Sewpersad

**Affiliations:** School of Engineering, Discipline of Chemical Engineering, University of KwaZulu-Natal, Durban 4000, South Africa; lokhat@ukzn.ac.za (D.L.); 215033639@stu.ukzn.ac.za (A.S.)

**Keywords:** dye, adsorption, carbon foam

## Abstract

The presence of dyes in effluents from textile industries has a detrimental effect on aquatic ecosystems as it hinders the process of photosynthesis by reducing the penetration of sunlight. The adsorption capacity of a carbon foam-based iron oxide sorbent obtained from natural sources for the removal of organic methylene blue (MB) dye from water was investigated. The adsorption capacities were examined by batch experiments, wherein the impacts of varying iron content, sorbent dosage, contact time, dye concentration, and characterization were assessed. The physical characteristics and surface morphology of the synthesized carbon foam were also investigated. The carbon precursor and iron oxide precursor were coalesced within a singular container and subjected to carbonization process. This resulted in the formation of a porous structure that is capable of effectively providing support to the iron oxide particles. The carbon foam produced is a self-assembled formation that possesses the characteristic shape and underlying network structure reminiscent of bread. As the number of nanoparticles went up, so did the number of active sites. At elevated temperatures, the interactions between the dye molecules were enhanced, resulting in a more efficient process of dye removal. The magnetite sample exhibited endothermic adsorption, and all other samples exhibited exothermic adsorption. The adsorption of MB onto iron supported by carbon foam did not exhibit intraparticle diffusion as the only rate-limiting step for all samples. The adsorption rate was governed by a multistep elementary reaction mechanism in which multiple processes occurred simultaneously. The experimental data in this study may be accurately modeled by the pseudo-second-order kinetic model (R^2^ > 0.96). Additionally, the Freundlich isotherm best describes the adsorption equilibrium, which is supported by the outstanding fit of data to the model (R^2^ > 0.999). The findings suggest that the utilization of a natural carbon foam as a support for an immobilized iron oxide sorbent demonstrates considerable effectiveness in the removal of methylene dye from industrial effluent.

## 1. Introduction

The release of hazardous wastes, poisonous gases, and smoke from industry has had a significant impact on the environment because of rapid industrialization and its products [1]. Numerous sectors, including textiles, paper, printing, plastic, food, and cosmetics, employ dyes to color their products [2]. This industry’s waste typically contains several colors that are dumped untreated into the drain water [3]. Due to the decrease in light transmission, dyes inhibit the photosynthetic movement of aquatic life [1]. In addition, some colors are toxic and carcinogenic to numerous aquatic organisms, such as fish and bacteria [4]. These dyes are responsible for serious harm to the kidneys, reproductive system, liver, brain, and central nervous system of humans [5]. Every year, people around the world use more than 10,000 different pigments and dyes that add color to things [6]. Due to the inherent non-biodegradability of most dyes, biological and chemical methods for dye removal have proven insufficient. Adsorption is currently being used for the removal of organic dyes due to its low cost, precision, viability, and easy design requirements [7]. Activated carbon is the preferred adsorbent for wastewater treatment, but its high cost restricts its industrial application [8]. Therefore, the textile industry is always looking for cheaper ways to replace activated carbon. 

The iron-based oxide nanomaterial exhibited a variety of sorption capabilities for concurrently removing colors, organic contaminants, and inorganic pollutants with great efficacy [6]. The environmental uses of iron oxide nanoparticles are strongly dependent on their features, including their magnetic properties, specific surface area, and core–shell structure [6]. Low toxicity, biocompatibility, and chemical inertness are three further benefits of iron oxide nanoparticles. 

Due to their increased specific surface area, high porosity, and strong magnetic response, nanoparticles based on iron exhibit remarkable sorption capabilities, resulting in an outstanding sorption capacity. Various types of iron oxide exist in nature, including *maghemite* (*gamma Fe*_2_*O*_3_), *magnetite* (*Fe*_3_*O*_4_), and *hematite* (*alpha Fe*_2_*O*_3_) [9]. Therefore, the stability of nanoparticles is essential for their high reactivity and mobility, as the rate of aggregation and deposition decreases with increasing material stability [6]. The preparation process of iron oxide nanoparticles is crucial in influencing particle shape and size, size distribution, surface chemistry, and therefore their applications [10]. 

The adsorption ability of an adsorbent is strongly influenced by its physicochemical features, including its porous structure, surface functional groups, and ion exchange capacity. Extensive research has been conducted on the chemical modification of adsorbents, which involves the use of acids, alkalis, and oxidants [11]. Physical modification, as an alternative approach, played a role in altering the structure, resulting in a more porous configuration that led to enhanced adsorption capabilities. 

Metal nanoparticles have been synthesized using a range of techniques, including the sol–gel process, chemical vapor deposition, chemical reduction method, solution-based synthesis, solvothermal method, reverse micelle method, and co-precipitation method [12].

Therefore, the present work aimed to examine the application of carbon foam as a substrate for iron nanoparticles for the purpose of dye adsorption on carbon foam derived from natural grains. Studies were carried out to assess whether this porous foam exhibited any thermal, mechanical, or sorption properties. The effects of the initial concentration, the effect of temperature, the effect of contact time, and the effect of the amount of carbon foam were investigated.

This study uniquely prepared the adsorbent by mixing the carbon precursor and iron oxide nanoparticle precursor and subjecting them to carbonization within a singular vessel. The simultaneous baking and carbonization of the natural grain results in the formation of a porous structure that effectively supports the iron oxide particles. 

## 2. Results

### 2.1. Sorbent Preparation

Utilizing the natural grain composition, carbon foam was created. For 5–10 min, the mixture was stirred with an electrically driven stirrer to ensure an even distribution of ingredients and a consistent texture throughout. The mixture was left to ferment at 60 °C for 60 min, baked at 180 °C for 40 min, and then 80 °C was used for 18 h to remove any extra water. The carbonization process took place in an argon gas atmosphere with a flow rate of 0.4 cm^3^/min, and the flow rate was controlled to provide a continuous flow of argon gas throughout the tube. On the tube furnace, the heating rate was set to 10 °C/min. Within the tube furnace, the carbonizing foam attained a maximum temperature of 600 °C and this temperature was maintained for 90 min (also known as the holding time). After 90 min, the carbonized foam in an argon environment had naturally cooled to 25 °C, which was the same temperature as the air around it. 

This procedure involves two chemical reactions: dehydration and carbonization. The following reaction occurs during carbonization in an argon gas atmosphere:

(C_6_H_10_O_5_) n (*starch*) → 6nC + 5nH_2_O (1)

The characteristics of porous carbon compounds are mostly determined by pore size and distribution. The density of carbon foam with bigger and irregular holes is low. By adjusting the quantities of yeast, water, iron nitrate, and magnetite in the mixture, the size of the foam’s holes can be altered. A few hundred micrometers in diameter, nearly spherical pores are visible to the human eye. 

In order to examine the thermal, mechanical, and absorbent properties of carbon foam, a total of five samples were synthesized. Samples A through D exhibited varying concentrations of iron and nitric acid, whereas the term “magnetite” denotes an addition of pure magnetite in the dry ingredients of the carbon foam precursor.

### 2.2. Characterization 

The addition of iron nitrate to granular carbon foam produced a structure that was more amorphous and porous. The material’s surface exhibited geometric characteristics, including an uneven shape, significant agglomerations, and a coarse texture, thereby offering additional sites for adsorption [13]. The transmission electron microscopy (TEM) micrographs of the natural grain carbon foam are presented in [14], which is part of our prior study. The results provide light on the pore structure of the carbon foam made from natural grains. The carbon foam has an increased surface area and micropore volume. The presence of iron enhances the action of the yeast, i.e., the consumption of sugars in the grain mixture and the production of carbon dioxide, which is responsible for developing the pore structure in the carbonized material [14]. 

### 2.3. Effect of Time 

**Figure 1:**
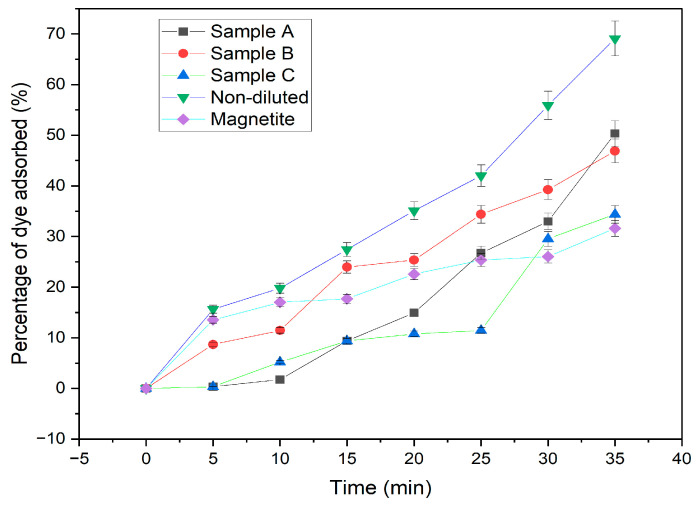
Dye removal using 40 mg/L solution under the effect of contact time.

The effect of contact time on adsorption at constant dye concentrations (MB—40 mg/L and 10 mg adsorbent) at different time intervals (5–35 min) was investigated, and the results are shown in Figure 1. The percentage of dyes removed rose as contact time increased [15]. The rate of adsorption was initially very high for magnetite, sample B, and D because the reactive site was still available on the surface of the adsorbent at the beginning of the adsorption process. Figure 1 indicates that sample D removes the largest amount of dye (70%) while sample C removes the least (35%) after 35 min, because the adsorption capacity of carbon foam was reduced after the nitric acid exceeded the optimum amount [16]. The results show that adsorption increases with time. The adsorption of dye was rapid for sample D compared to other samples. This rapid adsorption is due to iron and nitric acid being well-incorporated into the baking flour before the carbonization process [15]. For the first 10 min, the adsorption process was relatively low; thereafter, it increased steadily. In samples A, B, and C and in the magnetite sample, fluctuations in the percentage of dye removed can be seen. 

### 2.4. Effect of Sorbent Dosage Nanoparticles 

**Figure 2:**
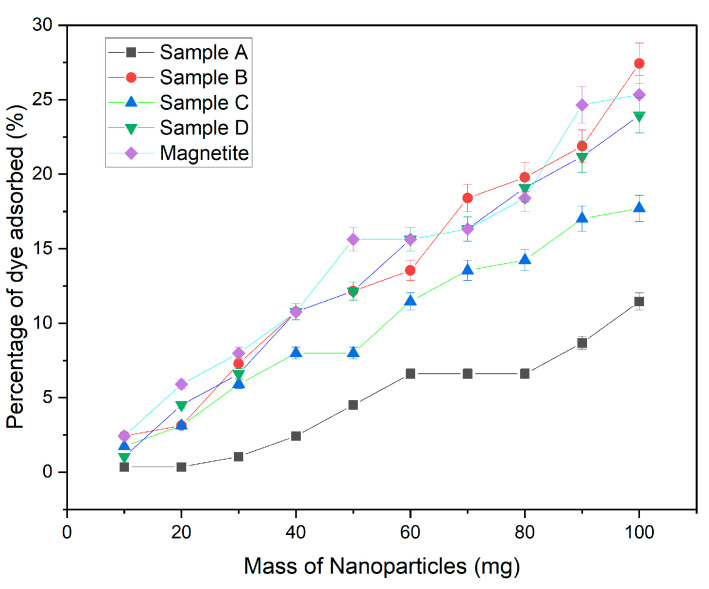
Dye removal using a varying number of nanoparticles in increments of 10 mg.

The number of nanoparticles containing iron added to each solution is exactly equal to the amount of dye removed. This merely indicates that as the quantity of nanoparticles increases, so does dye adsorption [17]. Figure 2 demonstrates that sample B, sample D, and the magnetite sample have effective adsorption properties when introduced in large volumes. The highest percentage of dye adsorbed was roughly 27% for all three samples, whereas samples A and C had the lowest percentages of dye removed from the solutions, 11.5% and 17.7%, respectively. Sample A had the least adsorption, followed by sample C; this indicates that sample B had the optimal number of nanoparticles, which is greater than sample A but less than sample C. This demonstrates that the number of iron-based nanoparticles plays a crucial role in influencing the adsorption of the adsorbent. Sample B, as well as the undilute and magnetite samples, had greater concentrations of iron, nitric acid, and magnetite; hence, these samples demonstrate a significantly higher adsorption rate. The growing number of adsorption sites can be attributed to the increased surface area of iron-based nanoparticles and the carbon foam support added to the solution. 

### 2.5. Effect of Initial Concentration 

**Figure 3:**
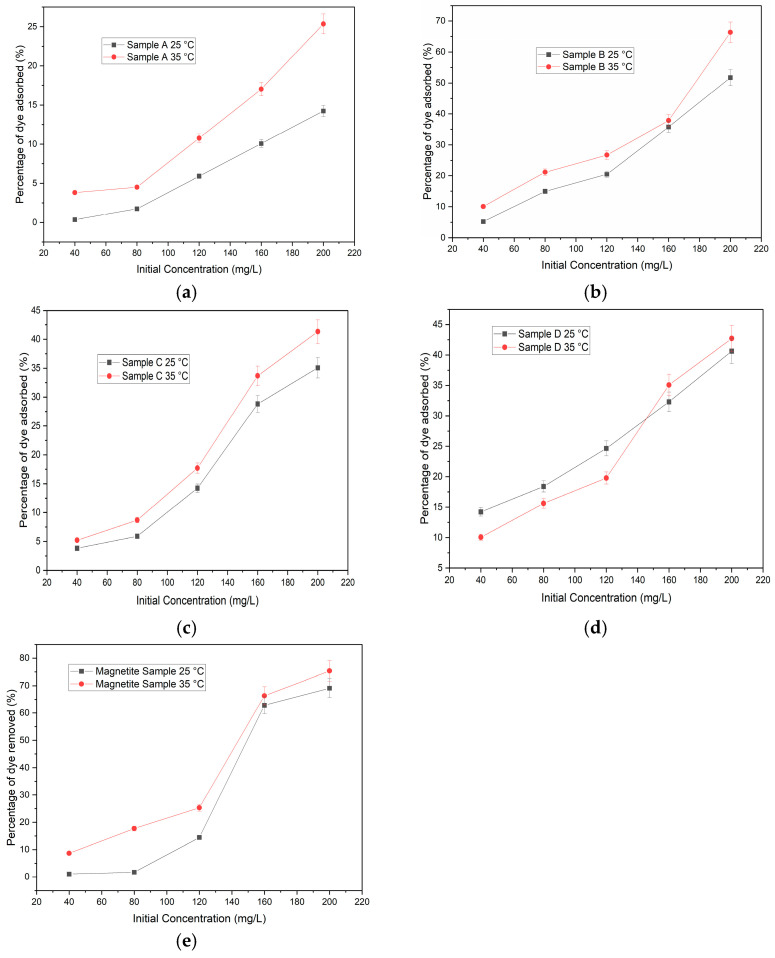
Sample temperature effects. (**a**) sample A, (**b**) sample B, (**c**) sample C, (**d**) sample D, and (**e**) magnetite sample. The study was conducted in two different temperatures, and 20–200 mg/L of MB.

Various initial dye concentrations (20–200 mg/L for MB) during the adsorption process at a given dosage of 10 mg of carbon foam were weighed and then added to 20 mL of solution, which was then agitated at 500 rpm for 10 min. Figure 3a–e demonstrates that an increase in the initial dye concentration induces an acceleration of the dye adsorption process [7]. The particles were magnetically drawn to the bottom of the beaker, and the remaining solution was added to the photocell of the spectrophotometer to produce a concentration reading. The test was conducted on all samples using the variously prepared solutions at room temperature. Figure 3 demonstrates that sample B, together with sample D and the magnetite sample, once again outperformed samples A and C, which exhibited significantly less effective adsorption properties. The maximum percentage of dye removed by samples B and magnetite was 24%, a substantially greater proportion than samples A and C, which removed 7.29% and 3.88% of the dye, respectively. In Table 1, all samples exhibited a modest percentage of dye removal for the first three solutions. However, for the last two solutions, samples B and magnetite exhibited a substantial increase. This indicates that these samples can remove dye from solutions with high concentrations [15]. 

### 2.6. Effect of Temperature 

Figure 3 depicts the effect of temperature on the adsorption rate, and it was discovered that as the temperature increased, so did the proportion of MB that was removed. An increased temperature increases the availability of active sites on the surface and the rate of pore volume opening in the adsorbent [6]. The material’s surface exhibited geometric characteristics, including an uneven shape, significant agglomerations, and a coarse texture, thereby offering additional sites for adsorption. In addition, as the temperature rises, the dye molecules exert kinetic energy, which increases the adsorption rate. 

The interaction between the dye molecules and the adsorbent should improve as the temperature rises, leading to an increase in the diffusion rate of MB dye molecules across the external boundary layer and internal pores of the iron-based nanoparticles [1]. To test this notion, a simple temperature test was conducted at 25 °C and 35 °C to discover if temperature has any effect on the amount of dye eliminated. Figure 3 demonstrates that every sample was tested at both temperatures. The magnetite sample exhibited the highest percentage of dye absorbed at both temperatures, followed by samples B, D, C, and A, respectively. Figure 3a,d,e demonstrate that performance is enhanced at both temperatures, demonstrating a straight proportional relationship. This proves the above-mentioned idea that a higher temperature promotes more absorption. All samples exhibit a rapid increase in adsorption at 35 °C when compared to the samples at 25 °C. The correlation between adsorption capacity and temperature indicates that the adsorption process requires heat to function efficiently. The results show that there might be a way to make a cheap and safe iron-based nanomaterial with a carbon foam support for use in industry, protecting the environment and making energy use more efficient. 

### 2.7. Equilibrium Adsorption 

The equilibrium state is achieved when adsorbate molecules encounter an adsorbent surface. Adsorption isotherms depict the state of equilibrium between the amount of adsorbate that is adsorbed (q_e_) and the concentration of the remaining adsorbate (C_e_) at a consistent temperature. Adsorption isotherms provide information on affinity, binding energy, adsorption capacity, and surface phase, which may be monolayer or multilayer. Modeling adsorption isotherms involves summarizing exponential data using theoretical or empirical equations and estimating isotherm parameters to compare adsorbent performance. The adsorption isotherm examines the relationship between dye absorption and dye concentration [18]. The empirical Freundlich model, which is derived from sorption on a heterogeneous surface and is suitable for low concentrations, can be expressed by the following equation:

(2) $${q_{e}}_{\;}=K_{F}{C_{e}}^{1/n}$$

C_e_ (mg/L) represents equilibrium adsorbate concentration, and q_e_ (mg/g) represents equilibrium adsorption quantity. K_F_ and n are proportional to adsorption capacity and intensity. Freundlich’s model is an empirical equation based on the solid–liquid solute equilibrium. The Freundlich model can describe heterogeneous surfaces but not adsorption data [19]. The software program Origin Lab 2023b was employed to estimate the isotherm parameters by a nonlinear regression analysis, specifically by fitting the relationship between the equilibrium adsorption quantity (q_e_) and the equilibrium concentration (C_e_). The Temkin model illustrates the interactions between adsorbate and adsorbent in an adsorption isotherm, specifically focusing on indirect interactions. The decrease in adsorption heat with coverage is expected to occur in a linear fashion because of the adsorbate–adsorbent interactions. Adsorption is characterized by a constant range of binding energies [18]. Temkin model equation:

(3) $${q_{e}}_{\;}=\frac{RT}{b}ln(AC_{e})$$

The variable q_e_ (mg/g) denotes the quantity of adsorption that occurs at equilibrium. C_e_ (mg/L) indicates the concentration of the adsorbate at equilibrium. T (K) is the temperature in Kelvin. R (J/mol/K) is the universal gas constant. Lastly, b (J/mol) corresponds to the Temkin model constant.

Table 2 displays the Freundlich R^2^ values determined by nonlinear fitting. Freundlich isotherm best represents the adsorption of MB, which indicates multilayer adsorption on a heterogeneous surface with variable energy distribution. n measures adsorption intensity. 1/n between 0 and 1 suggests MB adsorption [20].

### 2.8. Thermodynamic Analysis 

The thermodynamic parameters, Gibbs energy (∆G), enthalpy (∆H), and entropy (∆S), are key parameters that are utilized to assess the practical applicability of the adsorption process. The prediction of the spontaneously occurring process can be made in accordance with the values assigned to these parameters. The thermodynamic parameters were calculated utilizing the equations shown below [21]:

(4) ∆G=∆H−T∆S

(5) $$K_{c}=\frac{q_{e}}{C_{e}}$$

(6) $$lnK_{c}=\frac{∆S}{R}-\frac{∆H}{RT}$$

∆G = −RTlnK_c_ (7)

where K_c_, R, and T denote the equilibrium constant, the equilibrium concentration of dye in the solution, the equilibrium concentration of dye on the adsorbent, the gas constant (8.314 J/mol K), and the absolute temperature, respectively, (K). ∆H and ∆S values can be estimated using the slopes and intercepts of a graph depicting ln Kc vs. 1/T. As shown in Table 3 thermodynamic parameters can be determined using various equations and graphs. Temperature-dependent adsorption data are required for this purpose. R^2^ values (R^2^ = 1) indicate which plot is more appropriate for estimating the thermodynamic properties of the adsorption process. Thermodynamic investigations can reveal the spontaneity (∆G < 0), endothermic nature (∆H > 0), or exothermic nature (∆H < 0) of the adsorption process. The Gibbs free energy change serves as an indicator of the spontaneity and feasibility of adsorption processes across all samples. All the samples had an exothermic adsorption, except the magnetite sample, which had an endothermic adsorption. Table 3 depicts the intercepts and slopes of ln kc vs. 1/T graphs.

### 2.9. Adsorption Kinetics 

#### Pseudo-First and Pseudo-Second Order

For the research of adsorption kinetics modeling, Lager Gren pseudo-first-order and pseudo-second-order models were utilized. The following equation represents the pseudo-first-order nonlinear form:

q_t_ = (1 − e^−K1t^) (8)

The following equation represents the pseudo-second order in its nonlinear version.

(9) $$q_{t}=\frac{{q_{e}}^{2}K_{2}t}{1+K_{2}q_{e}t}$$

**Figure 4:**
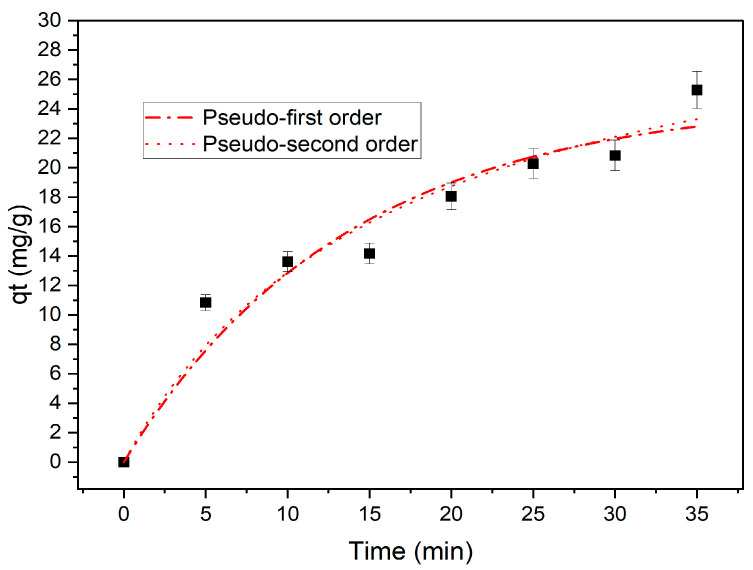
Magnetite sample fitting of pseudo-first order and pseudo-second order.

In this context, q_t_ (mg/g) represents the absorbed quantity at time t, q_e_ (mg/g) denotes the remaining quantity after reaching adsorption equilibrium, and K_1_ and K_2_ refer to the rate constants of the pseudo-first- and pseudo-second-order models, respectively. These rate constants are expressed in units of min^−1^ and g/mg/min. Shown in Table 4 are the calculated K_1_, K_2_, and q_e_ values as well as the corresponding linear coefficient of regression R^2^ values. Figure 4 depicts the application of pseudo-first-order and pseudo-second-order models for fitting purposes. The correlation coefficient R^2^ and the agreement between the calculated and experimental q_e_ values indicate the applicability of the model. Therefore, the pseudo-first-order model is predominant compared to the pseudo-second-order model. 

### 2.10. Intraparticle Diffusion 

The intraparticle diffusion resistance was calculated using Weber and Morris’s [22] intraparticle particle diffusion model, denoted by the following equation:

q_t_ = k_id_ t^1/2^ + c (10)

**Figure 5:**
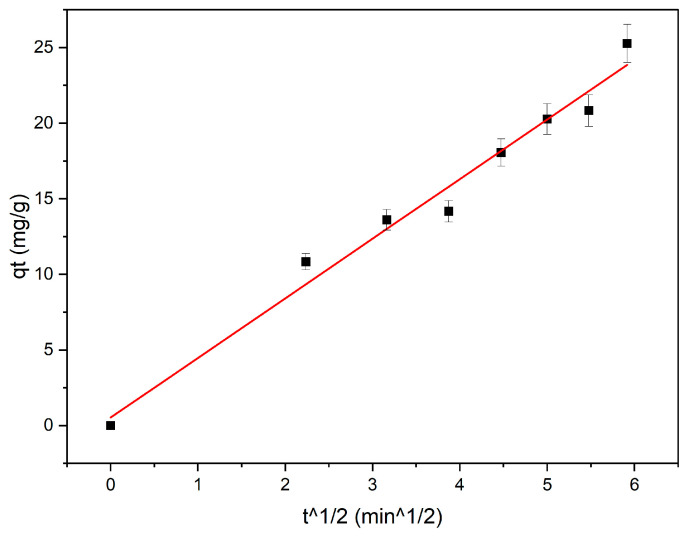
Intraparticle diffusion for the magnetite sample.

The variable q_t_ (mg/g) represents the quantity of adsorption at a given time t (min), while k_id_ (mg/g/min^1/2^) is the intraparticle diffusion model’s adsorption rate constant. Additionally, the parameter c is associated with the thickness of the boundary layer. If the relationship between the quantity of adsorbate (q_t_) and the time’s square root (t^1/2^) exhibits a linear pattern and intersects the origin, it can be inferred that the adsorption process is only governed by a single rate-limiting step. The rate is controlled by intraparticle diffusion when the origin is crossed by the uptake lines. Once the plots exhibit deviation from passing through the origin, it indicates that intraparticle diffusion alone is not the sole limiting factor in the rate of adsorption. This shows the presence of additional kinetic models that may influence the adsorption rate [22]. Figure 5 depicts the linear plot of intraparticle diffusion that passes through the origin. This finding indicates that intraparticle diffusion was not the sole factor restricting the rate of MB adsorption on all samples. Consequently, it is possible that other mechanisms could govern the adsorption rate for the other four samples [23]. 

## 3. Materials and Methods

### 3.1. Equipment and Materials Used

The following materials were used: 1000 g of baking flour, 50 g of dry yeast, water (distilled), magnetite, nitric acid, iron sponge, argon gas, nickel grease, pressurized air, and ethylene blue dye (powder). All chemicals were used as supplied with no further purification.

### 3.2. Sample Preparation

Five samples were created to investigate if carbon foam possesses thermal, mechanical, or absorbent qualities. The first four samples included variable amounts of iron and nitric acid, but the fifth sample contained a particular amount of magnetite. In this experiment, the amount of iron and nitric acid injected were among the test variables. The magnetite sample was prepared: 10 g of magnetite (iron sponge) were combined with 300 g of flour, 5 g of dry yeast, and 200 milliliters of water. Due to the somewhat larger size of the magnetite particles, a greater quantity of wheat and yeast was employed to accommodate them. The mixture was agitated at 500 rpm for 5–10 min. After preparing the five mixes, each mix was placed on an aluminum baking sheet to undergo the carbonization process. 

## 4. Conclusions

The findings of this investigation demonstrate that the adsorption capacity of a low-cost iron-based adsorbent for the removal of MB is significantly higher at 24 mg/g compared to the values of 6.7, 11.86, 17.3, and 17.5 mg/g reported in previous studies [24,25,26,27]. A systematic approach was utilized with a combination of literature research and experimentation methods to investigate iron-based nanoparticles in conjunction with a natural grain mixture procedure to determine any thermal, mechanical, and sorption properties that the particles may possess. Four tests were performed, namely the initial concentration and temperature tests, the effect of contact time, and varying the number of nanoparticles. The role of pore structure on temperature distributions has been examined; in particular, how pore structure affects the heat transferred throughout the material. Pore sizes can be varied by controlling the iron, nitric acid, magnetite, yeast, and water content. This allows the nanomaterials to be used for a variety of applications. The method of preparing the iron-based nanoparticles plays a key role in determining the particle shape and size, size distribution, active sites, and, subsequently, the applications. Samples B, D, and magnetite showed good adsorption properties as compared to samples A and C in all the tests performed. The Gibbs free energy of change indicates the spontaneity and practicability of adsorption processes for all the samples. All the samples had an exothermic adsorption, except the magnetite sample, which had an endothermic adsorption. When the temperature rises, the viscosity of the dye suspension reduces, allowing more adsorbate to permeate through the outer boundary layer and through the internal pores of the adsorbent. The adsorption process of MB onto iron supported by carbon foam was not solely governed by rate-limiting intraparticle diffusion. Instead, the adsorption rate was determined by a complex, multistep elementary reaction mechanism wherein multiple processes operated concurrently. The pseudo-second-order kinetic model is the best fit to the research in question, with (R^2^ > 0.96). Similarly, the adsorption equilibrium is best characterized by the Freundlich isotherm (R^2^ > 0.999). Based on the findings, it can be concluded that the utilization of iron oxide sorbent immobilized on natural carbon foam demonstrates high efficacy in the removal of methylene dye.

## Figures and Tables

**Table 1:** Initial concentration test.

Percentage of Dye Removed (%)					
Initial Concentration (mg/L)	A	B	C	D	Magnetite
40	0.35	1.74	0.35	0.35	5.21
80	2.43	3.82	0.35	4.51	5.90
120	8.68	1.04	1.04	8.68	8.68
160	4.51	14.24	3.13	10.07	18.40
200	7.29	23.29	3.82	12.15	23.96

**Table 2:** Freundlich isothermal equilibrium parameters.

Samples	R^2^
A	0.999
B	0.999
C	0.999
D	0.999
Magnetite	0.999

**Table 3:** Thermodynamic parameters.

Parameters	Sample A	Sample B	Sample C	Sample D	Magnetite
∆H	−334.80	−625.61	−334.80	−240.32	531.99
∆S	41.93	13.12	41.93	42.28	44.88
∆G	−12,829.59	−4534.47	−12,829.59	−12,840.51	−12,843.63

**Table 4:** Pseudo-first-order and pseudo-second-order parameters.

	Pseudo-First Order		Pseudo-Second Order	
Sample	K_1_	R^2^	K_2_	R^2^
A	6.70 × 10^−2^	0.985	1.15 × 10^−9^	0.848
B	4.50 × 10^−3^	0.986	4.89 × 10^−6^	0.986
C	6.28 × 10^−2^	0.945	2.00 × 10^−9^	0.825
D	1.04 × 10^−4^	0.977	3.32 × 10^−8^	0.976
Magnetite	7.34 × 10^−2^	0.985	2.19 × 10^−3^	0.955

## Data Availability

Data is contained within the article.
